# Supplementary material for: The role of V3 neurons in speed-dependent interlimb coordination during locomotion in mice
Source: eLife. 2022 Apr 27;11:e73424. doi: 10.7554/eLife.73424 (PMC9045817; doi:10.7554/eLife.73424)
Supplement: Supplementary file 1. [file elife-73424-supp1.docx]

**Statistical results**

| Figure 1 C3 | RC5 | | | LC5 | | | RC8 | | |
| --- | --- | --- | --- | --- | --- | --- | --- | --- | --- |
|  | Ringer | KA | WO | Ringer | KA | WO | Ringer | KA | WO |
| Mean | 2.029 | 1.722 | 1.938 | 2.053 | 1.745 | 1.943 | 1.789 | 1.839 | 1.744 |
| P value | 0.1072 | | | 0.1076 | | | 0.7573 | | |
|  | LC8 | | | RL5 | | | LL5 | | |
|  | Ringer | KA | WO | Ringer | KA | WO | Ringer | KA | WO |
| Mean | 2.063 | 1.721 | 1.988 | 2.526 | 0.9832 | 1.271 | 2.536 | 1.146 | 2.168 |
| P value | 0.1102 | | | 0.0000 | | | 0.0000 | | |

| Figure 2 C | Resting | 15 cm/s | 40 cm/s |
| --- | --- | --- | --- |
| Mean (%) | 2.056 | 20.2 | 57.79 |
| P value | Resting Vs 15 cm/s | Resting Vs 40 cm/s | 15 cm/s Vs 40 cm/s |
|  | 0.0495 | 0.0162 | 0.0174 |

| Figure 3 | WT | V3^OFF^ |
| --- | --- | --- |
| Mean (cm/s) | 76.43 | 34.09 |
| P value | 0.00000 | |

| Figure 4  A1-D1 | Homologous-hind | | Homologous-fore | | Homolateral | | Diagonal | |
| --- | --- | --- | --- | --- | --- | --- | --- | --- |
|  | WT | V3^OFF^ | WT | V3^OFF^ | WT | V3^OFF^ | WT | V3^OFF^ |
| Mean | 0.494 | 0.490 | 0.518 | 0.510 | 0.450 | 0.343 | 0.970 | 0.845 |
| P value | 0.19330 | | 0.06134 | | 0.00000 | | 0.00000 | |
|  |  | |  | |  | |  | |
| Kappa | 4.418 | 2.600 | 2.754 | 2.718 | 3.600 | 3.408 | 3.098 | 2.674 |
| P value | 0.00000 | | 0.77626 | | 0.23034 | | 0.00136 | |

| Figure 4 A2-D2  Mean and P value | | Homologous-hind | | | Homologous-fore | | | Homolateral | | | Diagonal | |
| --- | --- | --- | --- | --- | --- | --- | --- | --- | --- | --- | --- | --- |
|  |  | WT | V3^OFF^ | WT | | V3^OFF^ | WT | | V3^OFF^ | WT | | V3^OFF^ |
| 15 cm/s | Mean | 0.492 | 0.480 | 0.522 | | 0.503 | 0.460 | | 0.327 | 0.979 | | 0.830 |
|  | P value | 1.00000 | | | 0.32552 | | | 0.00000 | | | 0.00000 | |
| 20 cm/s | Mean | 0.497 | 0.483 | 0.510 | | 0.495 | 0.445 | | 0.331 | 0.952 | | 0.826 |
|  | P value | 0.48704 | | | 0.90003 | | | 0.00000 | | | 0.00000 | |
| 25 cm/s | Mean | 0.495 | 0.490 | 0.519 | | 0.505 | 0.431 | | 0.334 | 0.953 | | 0.833 |
|  | P value | 1.00000 | | | 0.93150 | | | 0.00000 | | | 0.00000 | |
| 30 cm/s | Mean | 0.497 | 0.496 | 0.525 | | 0.525 | 0.442 | | 0.340 | 0.972 | | 0.859 |
|  | P value | 1.00000 | | | 1.00000 | | | 0.00000 | | | 0.00000 | |
| 35 cm/s | Mean | 0.488 | 0.479 | 0.520 | | 0.515 | 0.449 | | 0.374 | 0.970 | | 0.861 |
|  | P value | 1.00000 | | | 1.00000 | | | 0.00000 | | | 0.00000 | |
| 40 cm/s | Mean | 0.498 | 0.573 | 0.513 | | 0.560 | 0.473 | | 0.432 | 0.989 | | 0.980 |
|  | P value | 0.00001 | | | 0.00367 | | | 0.00478 | | | 1.00000 | |

| Figure 4 A2-D2  Variability (concentration parameter kappa) and P value | | Homologous-hind | | | | Homologous-fore | | | Homolateral | | | | Diagonal | | |
| --- | --- | --- | --- | --- | --- | --- | --- | --- | --- | --- | --- | --- | --- | --- | --- |
|  |  | WT | | V3^OFF^ | WT | | V3^OFF^ | WT | | V3^OFF^ | | WT | | | V3^OFF^ |
| 15 cm/s | Kappa | 2.839 | 4.408 | | | 2.463 | 3.454 | | 2.890 | | 4.603 | | 2.336 | 4.695 | |
|  | P value | 0.00053 | | | | 0.01221 | | | 0.00023 | | | | 0.00000 | | |
| 20 cm/s | Kappa | 4.655 | 3.515 | | | 2.465 | 2.939 | | 3.605 | | 3.618 | | 2.786 | 3.771 | |
|  | P value | 0.10317 | | | | 0.59857 | | | 1.00000 | | | | 0.02673 | | |
| 25 cm/s | Kappa | 4.587 | 2.332 | | | 2.431 | 2.900 | | 3.759 | | 3.222 | | 3.098 | 2.553 | |
|  | P value | 0.00000 | | | | 0.56653 | | | 0.94629 | | | | 0.45078 | | |
| 30 cm/s | Kappa | 5.561 | 2.927 | | | 2.797 | 2.965 | | 4.669 | | 3.795 | | 3.821 | 2.803 | |
|  | P value | 0.00000 | | | | 1.00000 | | | 0.44578 | | | | 0.02580 | | |
| 35 cm/s | Kappa | 5.301 | 1.682 | | | 3.023 | 1.838 | | 4.490 | | 3.030 | | 4.017 | 1.906 | |
|  | P value | 0.00000 | | | | 0.00036 | | | 0.00815 | | | | 0.00000 | | |
| 40 cm/s | Kappa | 5.050 | 1.424 | | | 3.688 | 2.225 | | 3.255 | | 3.898 | | 3.351 | 1.875 | |
|  | P value | 0.00000 | | | | 0.00372 | | | 1.00000 | | | | 0.00069 | | |
